# Income-Generating Processes of Free Web-Based Digital Health Tools to Engage Patients: Qualitative Analysis

**DOI:** 10.2196/23654

**Published:** 2021-02-03

**Authors:** Claudia Lai, Raisa Deber, Alejandro R Jadad, Aviv Shachak

**Affiliations:** 1 Institute of Health Policy, Management and Evaluation Dalla School of Public Health University of Toronto Toronto, ON Canada; 2 Department of Anaesthesiology and Pain Management Faculty of Medicine University of Toronto Toronto, ON Canada; 3 Faculty of Information University of Toronto Toronto, ON Canada

**Keywords:** digital health, patient engagement, eHealth, health information

## Abstract

**Background:**

In recent years, digital tools have become a viable means for patients to address their health and information needs. Governments and health care organizations are offering digital tools such as self-assessment tools, symptom tracking tools, or chatbots. Other sources of digital tools, such as those offered through patient platforms, are available on the internet free of charge. We define patient platforms as health-specific websites that offer tools to anyone with internet access to engage them in their health care process with peer networks to support their learning. Although numerous social media platforms engage users without up-front charges, patient platforms are specific to health. As little is known about their business model, there is a need to understand what else these platforms are trying to achieve beyond supporting patients so that patients can make informed decisions about the benefits and risks of using the digital tools they offer.

**Objective:**

The aim of this study is to explore what patient platforms are trying to achieve beyond supporting patients and how their digital tools can be used to generate income.

**Methods:**

Textual and visual data collected from a purposeful selection of 11 patient platforms from September 2013 to August 2014 were analyzed using framework analysis. Data were systematically and rigorously coded and categorized according to key issues and themes by following 5 steps: familiarizing, identifying a thematic framework, indexing, charting, and mapping and interpretation. We used open coding to identify additional concepts not captured in the initial thematic framework. This paper reports on emergent findings on the business models of the platforms and their income-generating processes.

**Results:**

Our analysis revealed that in addition to patients, the platforms support other parties with interests in health and information exchanges. Patient platforms did not charge up-front fees but generated income from other sources, such as advertising, sponsorship, marketing (eg, sending information to users on behalf of sponsors or providing means for sponsors to reach patients directly), supporting other portals, and providing research services.

**Conclusions:**

This study reports on the mechanisms by which some patient platforms generate income to support their operations, gain profit, or both. Although income-generating processes exist elsewhere on social media platforms in general, they pose unique challenges in the health context because digital tools engage patients in health and information exchanges. This study highlights the need to minimize the potential for unintended consequences that can pose health risks to patients or can lead to increased health expenses. By understanding other interests that patient platforms support, our findings point to important policy implications, such as whether (and how) authorities might protect users from processes that may not always be in their best interests and can potentially incur costs to the health system.

## Introduction

In recent years, digital tools have become a viable means for patients to address their health and information needs. Governments and health care organizations worldwide are offering digital tools to engage patients, such as self-assessment tools [1], apps to track daily symptoms [2], or chatbots to provide questions and answers [3]. In addition to government and health care organizations, digital tools, such as those found on patient platforms, are now freely available on the internet. Patient platforms are defined as health-specific websites that offer virtual tools to engage patients in their health care process, with peer networks to support user learning (eg, PatientsLikeMe, WebMD). Virtual tools can support the sharing of information that would otherwise be difficult to access, such as personal health information and experiences shared by other patients, thereby enabling some patients to actively learn virtually anywhere at any time, and to receive and provide peer support [4].

Despite the potential benefits, at least for patients who prefer to take an active role in managing their health issues, patient platforms introduce new challenges because their digital tools are not subject to the same rules, regulations, and norms as those offered by health care providers or health care organizations. In contrast, digital tools offered by health care providers or health plans, for example, are governed by privacy legislations such as the *Health Insurance Portability and Accountability Act* in the United States or the *Personal Health Information Protection Act* in Ontario, Canada. In the United States, the US Food and Drug Administration (FDA) is addressing regulation for software as medical devices and regulates a small subset of health apps at this time, for example, those that pose a higher risk if they do not work as intended [5,6]. In other countries such as Canada, new approaches to regulate digital health technologies are not yet in place.

Although the majority of digital tools freely available on the internet from patient platforms are not governed by health authorities, they can influence how patients manage their health issues and whom they consult with for health care advice (eg, health care providers or other patients with related disease or drug use experience). A US survey reported that 1 in 5 users decided to stop medication and 6% to 21% changed their physician as a result of using a patient platform [7]. These actions could influence how patients manage health issues in ways that might benefit or potentially harm them. Two factors that may influence how patient platforms engage their users include stakeholders whose interests the platform serves, including, but not limited to, health care providers, pharmaceutical companies, and insurance companies, and the business model or income-generating mechanisms of the platform. With a growing number of patients using social media tools [4,8], understanding these issues can help patients make informed decisions about the benefits and risks of using patient platforms and inform policy makers on whether and how they might be regulated. Thus, the purpose of this study is to gain insights into what patient platforms are trying to achieve beyond supporting patients, what other stakeholders they support, and how they generate income.

## Methods

### Overview

A total of 11 patient platforms were purposefully selected, as described later. Textual and visual data collected from these platforms from September 2013 to August 2014 were analyzed using framework analysis [9]. Ethics approval was obtained from the University of Toronto Research Ethics Board.

### Platform Selection

Patient platforms were selected from academic literature, patient advocacy websites, industry websites, and internet searches using various search terms such as *social media platforms*, *patient tools*, and *online patient communities*. Platforms were chosen to partly capture variability across the following attributes: (1) for-profit and not-for-profit platforms; (2) platforms supporting single or multiple conditions; and (3) platforms supporting different disease dimensions, including rare conditions (as defined by the platform itself), mental health conditions, and progressive chronic conditions. Progressive chronic conditions were selected from 3 distinct types of illness trajectories, as described by Murray et al [10]: Trajectory 1, which refers to chronic conditions with short periods of evident decline (eg, incurable cancer); Trajectory 2, which refers to chronic conditions with long-term limitations and intermittent episodes of acute deterioration (eg, multiple sclerosis [MS]); and Trajectory 3, referring to chronic conditions with a prolonged gradual decline (eg, neurological conditions) [10]. We deliberately explored platforms offering tools in English (from the United States and Canada), which support patients who can have high but diverse health and information needs. This platform selection strategy allowed for the discovery of concepts and what platforms have in common and unique features in their situated context [11]. Table 1 describes the rationale for case selection using these platform dimensions.

**Table 1:** Platform dimensions guiding case selection.

Dimension	Platform selection	Rationale
Number of disease groups	Specific group of patients Multiple patient groups	Platforms that might achieve higher levels of patient engagement by pooling resources to support a specific group of target users versus platforms that might have rich resources to target a broader range of users
Disease-related characteristics	Rare disease (eg, amyotrophic lateral sclerosis) Nonprogressive conditions (eg, anxiety disorder, physical disability, diabetes) Progressive conditions (eg, Trajectory 1: steady progress and usually a clear terminal phase such as cancers; Trajectory 2: long-term limitations and gradual decline with intermittent episodes of acute deterioration and some recovery such as organ failure or multiple sclerosis; and Trajectory 3: prolonged gradual decline such as neurological conditions)	Platforms that might achieve higher levels of patient engagement by targeting potential users with high health and information needs

We made efforts to select platforms that provide rich information [12], such as platforms with high utilization (eg, number of registered users and user postings, site traffic as measured by industry tools such as Alexa Rank), and operating for long periods (website start date and date of user postings). Platforms with outdated content (ie, no new content in the last 6 months) or limited user postings were excluded from the analysis. This allowed us to explore how they might promote health and encourage information sharing. No additional platforms were added postanalysis because recurring patterns were discovered across the range of platforms [13], reaching saturation on a set of concepts. Table 2 presents the characteristics of the sample based on the aforementioned selection criteria.

**Table 2:** Characteristics of the selected platforms.

Platform	Single or multiple	Disease dimension	For profit or not for profit	Year started
A1	Single condition	Rare condition	Not for profit	2006
A2	Multiple conditions	Multiple related chronic conditions *Trajectory 3: prolonged dwindling*	Not for profit	1993
A3	Single condition	Chronic condition *Trajectory 1: short period of evident decline*	Not for profit	2000
A4	Multiple conditions	Multiple different conditions	For profit	2005
A5	Multiple conditions	Multiple different conditions	For profit	1994
A6	Single condition	Chronic condition *Trajectory 2: long-term limitations with intermittent episodes*	Not for profit	N/A
A7	Single condition	Multiple related chronic conditions *Trajectory 2: short period of evident decline*	For profit	2010
A8	Multiple conditions	Multiple different conditions	For profit	2004
A9	Multiple conditions	Multiple different conditions	For profit	N/A
A10	Single condition	Mental health condition	For profit	N/A
A11	Single condition	Mental health condition	For profit	2000

### Data Collection

Textual and visual data were collected from the platforms’ homepages and other webpages relating to the platforms. Because websites are dynamic and subject to changes in design, content, and link structures (both from and to the website), data were captured as screenshots, saved, and stored for offline analysis [14]. These included 302 documents and screenshots collected from September 2013 to August 2014, saved, and stored for offline analysis using the NVivo 11 qualitative data analysis software. Table 3 describes the types of data collected, such as frequently asked questions*, About Us* pages, descriptions of tools, mission statements, registration forms, terms of use, and privacy policies.

These sources offered rich data to analyze what the platforms were trying to achieve, how, and for whom. Only publicly available data that did not require site registration were collected for analysis. No specific platforms, platform users, user groups, or communities are identified in this publication by name. Although platforms may be identifiable, the names of the platforms are masked, as our goal is to illustrate broader issues that can occur on any patient platform and not to single out any individual platform or platform owner. Interested readers may contact the authors for a list of platforms.

**Table 3:** Data from website home pages and other webpages.

Data collected	Example of data collected	Total number of web pages or documents
Website home pages	Site purpose; platform characteristics (eg, target user groups, number of registered users); description of the platform tools	12
Frequently asked questions	Platform tools, funding sources, and rules pertaining to the website use	32
Site owner information	About us, site, site owners or investors, site mission, testimonials, news releases, and owner webpages	40
Registration process	Registration form	12
Sponsors and partners	Sponsors, partners, and promotional information for the sponsors and partners	37
Tools	Tools and description of tools	133
Terms of use	Policies relating to the use of the platform; how to dispute issues; how to remove user information or terminate account	11
Privacy policies	Policies relating to how user information are collected and used	11
Other rules or policies	Rules relating to forums, advertising and editorial policies, and other rules governing the platform use	5

### Data Analysis

Data were systematically tracked and analyzed using framework analysis, which offers a systematic procedure for *sifting, charting, and sorting material according to key issues and themes* [9] by following 5 steps: familiarizing, identifying a thematic framework, indexing, charting, and mapping and interpretation. Preliminary data were systematically and rigorously coded and categorized according to the initial thematic framework, as described elsewhere [15]. Open coding was used to identify additional concepts not captured in the initial thematic framework [16]. Data were subsequently grouped into additional categories, as new concepts were identified from the data. Finally, recurring and overarching themes, unique themes, relationships between various themes and other contextual factors were identified to form theoretical propositions. The coding of data and emergent themes were discussed at bimonthly work sessions with the first and last authors (CL, AS) to reduce potential bias that comes from a single researcher and to increase reliability in observations. Emergent themes were discussed at regular meetings with the research team, including all authors of this paper. This paper reports on concepts from the category of platforms’ business model and income-generating processes, which were identified through the aforementioned process of open coding outside of the initial framework.

## Results

### Platform Sponsors and Partners

Our analysis revealed that in addition to supporting patients, the platforms support other parties with interests in health and information exchanges on the patient platforms. These other parties include patient advocacy organizations, pharmaceutical companies, insurance companies, hospitals and health care providers, employers, health plan administrators, regulators and government organizations, corporations, foundations, and individual donors. Platforms referred to these other parties as *sponsors*, or *partners*, where the distinctions were not always clear; for example, *sponsors* referred to other parties who donated funds to the platform. However, *sponsors* can also provide or fund specific tools or purchase services such as advertising or marketing services. *Partners* referred to other parties, such as patient advocacy organizations or researchers who might collaborate with or without financial arrangements. However, *partners* can also provide tools or purchase services such as clinical trial recruitment services or marketing services. Table 4 provides a summary of sponsors and partners and their relationships with platforms.

**Table 4:** Partners and sponsors and their relationships with platforms.

Partners and sponsors	Relationship with platforms
Patient advocacy groups	Platforms owned by patient advocacy organizations (A3, A6); facilitated forums for patient advocacy groups (A4, A8) and supported patient advocacy platforms (A11)
Pharmaceutical companies, researchers, and insurance companies (eg, partners or stakeholders who may purchase data or research services)	Pharmaceutical companies, researchers, and other health companies (such as insurance companies) listed as partners or sponsors (A3, A4, A6, A7, A8, A9, A10, and A11)
Hospitals and health care providers (eg, health care providers who may purchase tools for their clients)	Hospitals and health care providers listed as partners and sponsors (A1, A7); hospitals and health care organizations described as clients (A5, A7, A9)
Employers, health plan administrators, regulators, and governments (eg, organizations that may purchase tools for a particular group of users)	Employers, health plan administrators, regulators, and *innovative government organizations* listed as partners or sponsors (A5, A7, A9, A11)
Corporations, foundations, and individual donors	Platforms seeking donations from corporations, foundations, and individual donors (A1, A3, A6)

### Income-Generating Mechanisms

All the platforms we studied offered tools free of charge and generated income in other ways, such as through advertisements, fundraising and donations, sponsored tools, marketing services, tools to support other platforms or portals, and research services. Examples are illustrated in Table 5 and described in detail later.

**Table 5:** Examples of data on income-generating processes.

Item	Category	Examples
a	Advertising	We also may use Behavioral Advertising cookies which are a way of providing advertisements on the websites you visit and making them more relevant to your interests. (A6)
b	Fundraising	Shop at AmazonSmile and Amazon will make a donation to [A1]: Get started (A1) Welcome to Shop for [A3]. Our partners have selected or created items specifically for the benefit of [A3]. A portion of the sale proceeds from your purchase of these items will be donated to [A3], which enables us to continue to provide our online resource to the millions of people we serve each year. Thank you for your contribution. (A3) Advocacy and Fund-Raising Fundraising Opportunities for [A3] 16 Topics 252 Posts Help support our organization, [A3]! Please note, this is NOT a forum for fundraising for other organizations. (A3)
	Donations	Thanks To Our Sponsors Want to sponsor this site? Corporate and individual sponsors welcome… Help us help… Help us change even more lives by donating spare change. (A1) Thank you for your donation! Here is a forum to share with others who you're honoring with your donation. (A3)
c	Sponsored tools	This dictionary is a compilation of numerous complex breast cancer terms defined in plain English. This program was developed by [A3], and is sponsored in part by an unrestricted educational grant from Bristol-Myers Squibb Oncology, and launched in partnership with... (A3) Start now: Each module below includes information to help you learn and manage your disease including: Videos, publications, worksheets, links to relevant web pages… [A6] gratefully acknowledges these educational grants to support this project. Bayer HealthCare. Biogen, Novartis, Sanofi Genzyme, Teva Neuroscience (A6)
d	Marketing Coupon to save money	We also provide a voluntary opt-in service to allow partners to directly communicate with patient members through our system. To learn more, see how we make money or read details on what is shared and sold in our Privacy Policy. (A8) We make managing chronic health conditions easier. We'll work with your doctor and insurance provider to get your medications delivered right to your door at little or no cost. Save Money Every Month. What if you could save money every single month on the prescription medications you already need and use?...No Insurance? No Problem. What if you could save money every single month on the prescription medications you already need and use? What if you could pay less for your prescribed medications, even without insurance? Believe it or not, such a thing is possible. But don’t take our word for it, get in touch with us and find out for yourself! (A10)
	Recruit patients	A diabetes pharma brand sought to increase awareness and patient enrollment through branded placement within targeted content on [a diabetes patient platform]. Their goal was to recruit patients at a cost of under $100 per patient enrollment. A blood pressure pharma brand presented a coupon to a narrow, qualified audience using our xxx program. Of more than 28k leads delivered, nearly 10% converted to a prescription, well over target A pharmaceutical diagnostics brand sought qualified leads through our xxx program. We delivered more than 300k qualified leads and 27k new prescriptions. The cost per new patient was $60 and offered a value of $280 to the brand. They quadrupled their [platform] lead budget the following year. (A10 site owner)
e	Support other portals	Important note for clinic patients. If you have been directed to [A7] by your clinic, do not join here. Instead, use the link that was sent to you via email, or sign up through your clinic's website. (A7)
f	Research	[A8] may also periodically ask Members to complete short surveys about their experiences (including questions about products and services). Survey responses are analyzed, combined with Members’ Shared Data and shared with and/or sold to Partners. Member participation in these surveys is not required, and refusal to do so will not impact a Member’s experience on the Site. (A8) We take the information patients like you share about your experience with the disease and sell it to our partners (ie, companies that are developing or selling products to patients). These products may include drugs, devices, equipment, insurance, and medical services. Except for the restricted personal information you entered when registering for the site, you should expect that every piece of information you submit (even if it is not currently displayed) may be shared with our partners and any member of [A8], including other patients. (A8)

#### Advertisements and Sponsored Content

Most patient platforms, including both for profit and not for profit, generated income by posting advertisements and sponsored content at prominent places on their website. Posting advertisements was framed as beneficial for users, such as “to provide you with our award-winning content at no cost to you” (A9), for the “convenience to patients” (A5), or for helping patients “make educated healthcare choices” (A10). Platforms also noted how they tailored advertisements delivered to their users (examples on behavior advertising are given in Table 5). Advertisements were typically labeled as such. For example, 1 platform noted how they took “steps to ensure that you can clearly identify content that is provided by and is under the editorial control of our sponsors before you view it, so you can make an informed decision as to whether or not to view it” (A9). However, this was not always the case. Platforms sometimes did not make a clear distinction between content intended for the benefit of patients (eg, evidence-based health information) and content intended to benefit sponsors (eg, advertisements or sponsored content). For example, the terms of use for platform A7 described “marketing material from pharmaceutical manufacturers and company information and data about cancer care” in the same sentence as *articles*, *news reports*, or *calculation tools* intended to educate patients. In another case, platform A10 marketed its editorial page for posting sponsored content, as noted below:

Sponsorship

Highly marketable full editorial page –your content or ours.

A10 owner site

LEVERAGE OUR BRAND TRUST

Let us introduce you to our users with a custom performance-based campaign.

A10 owner site

#### Fundraising, Individual, and Corporate Donations

Three platforms (A1, A3, and A6), which operated not for profit, sought financial support through fundraisers, individual donations, corporate sponsorships, and grants. These platforms were condition-specific platforms and sought funds to support a rare disease (A1), cancer (A3), or chronic condition (A6). For example, we found instances where platforms asked users to shop at designated retailers from which the platform receives rewards for purchases (A1), buy mugs or products of which a portion of the sales are donated to the platform (A3), or participate in forums dedicated to fundraising opportunities (A3), as illustrated in Table 5. Donations were also sought from individual donors or corporate sponsors, where sponsors are acknowledged on the platform. We also found tools created from sponsorships or grants, which are described later.

#### Sponsored Tools or Disease Awareness

Five platforms (A1, A3, A4, A9, and A10), including both for-profit and not-for-profit platforms, provided users with sponsored tools, referring to tools produced or funded by platform sponsors or partners, such as pharmaceutical companies. Sponsored tools included tools peer reviewed by clinicians, tools produced through *educational grants*, or tools for disease awareness, as illustrated in Table 5, sponsored tools. The following is an example of a sponsored self-assessment tool funded by 2 pharmaceutical companies that sell drugs to treat MS:

Multiple Sclerosis Assessment

This content is selected and controlled by [A9’s] editorial staff and is brought to you by EMD Serono, Inc. and Pfizer Inc.

How Well Are You Managing MS?

… Answer a few questions, and you'll get:

Treatment options for your type of MS

Information about the progression of the disease

Tips for dealing with symptoms while still enjoying life

Reviewed by [name]MD on [A9] (A9)

Sponsored tools were presented as being mutually beneficial for sponsors who might also be interested in raising disease awareness.

#### Marketing Services

Seven platforms (A3, A4, A6, A7, A8, A9, and A10), including both for-profit and not-for-profit platforms, generated income by providing marketing services (Table 5). Users were often asked to consent to receiving marketing material to “inform you of other offers, services, or websites available from [A10] or third parties including our advertising partners” (A10). In some cases, the platforms provided sponsors with a means “to directly communicate with patient members through our system” (A8). Users were sometimes required to provide implicit consent for the sharing of their personal information with site sponsors during the site registration process or when they registered for using specific platform tools, such as patient communities, as noted below:

When you register to join a [A10] community and/or register for offers available through our advertising partners, you consent to sharing information about yourself, Personally Identifiable Information, so that we can make our services and the services of our partners available to you.

A10

In addition, some platforms generate income by recruiting patients, such as for clinical trials. For example, platform A4 described how they help patients “ﬁnd relevant clinical trials by inviting them to connect with researchers seeking qualiﬁed participant[s],” where the “clinical trial sponsors pay” platform A4 for the service. Besides clinical trial recruitment, 1 platform generated income by providing coupons to users, which was marketed to help them “save money on their healthcare costs” (A10). On the platform owner’s website, their services were marketed to sponsors for recruiting patients to change medical interventions to those of the platform’s sponsors (eg, blood pressure and diabetes medications or diagnostic products), as illustrated in Table 5.

#### Tools to Support Health Portals or Other Platforms

Three platforms (A7, A9, and A11), which operated for-profit platforms, marketed their tools (or services) to support the health portals of other organizations. For example, platform A7 advised “clinic patients” to sign up, or login using “the link that was sent to you via email or sign up through your clinic's website,” as noted in Table 5. Platform A9 described offering their external users with additional services, such as “online and offline health risk assessments, lifestyle education and telephonic health coaching” (A9). Similarly, platform A11 described how their publicly available patient platform offered tools to engage users free of charge for the purpose of *beta-testing software* (eg, testing software updates or new tools), which can later be marketed to other organizations, such as not-for-profit organizations, or pharmaceutical companies.

#### Research Services

Two platforms (A8 and A9), which operated for-profit platforms, generated income by supporting research services in 1 of the 2 ways. First, platforms described how they conducted surveys or questionnaires for other parties, as illustrated in Table 5. Beyond surveys or questionnaires, 1 platform (A8) described how they are available to support the writing of grant proposals or protocols for review boards, as illustrated below:

We are proud to collaborate with some of the leading research institutions in the world on useful and interesting academic research. Please write to the research team with your initial research proposal. If we think a research project has the potential to benefit our users, we would be happy to assist you in writing a grant proposal and helping to describe what we do for your local Internal Review Board (IRB). The proportion of funding we would receive depends on a number of factors including the contribution of our staff to the design, the difficulty of accessing the specific population of interest, and the source of funding.

A8

Second, platforms collected a repository of user-generated health data through the platform itself, such as through user profiles or self-tracking tools, as illustrated below:

Track your healthcare

Chart your health over time and contribute to research that can advance medicine for all.

A8

How is the optional background information used? The more other users know about the users rating the drugs, the more valuable those ratings become. An 18-year-old college student might have a different experience with a particular medication than a post-menopausal woman of 60 on the same drug. Once we have a good sampling of this data, we’ll begin letting you search the effectiveness of a drug in individuals similar to you.

A9

Although the repository of data can support other users doing research for themselves, 1 platform (A8) was transparent in disclosing their intention to sell the information to “companies that are developing or selling products to patients” which included companies that sell “drugs, devices, equipment, insurance, and medical service” (A8), as noted in Table 5. The information users were required to share included their health profile, biographical information, condition or disease information, treatment information, symptoms, health scores or charts over time, laboratory results, and *connections with other people* on the platform. In fact, this platform advised users to expect *almost any piece of information they submitted*, including information which may not be accessible to the users themselves (or *not currently displayed*), to be shared or sold (except restricted data such as their email address, name, and physical address). This platform (A8) was the only one transparent about this and had even warned users of the potential risks of sharing information online:

When sharing information online about your health or a specific condition, you should know there is always a risk that someone could use this information against you. For example, medical and life insurance companies have clauses that exclude pre-existing conditions or employers may not want to employ someone with a high-cost or high-risk disease. We know these risks are real.

A8

## Discussion

### Overview

Despite the introduction of new ways for patients to participate in their health care process and the growing availability of institutional patient-oriented health information systems such as patient portals [17-19], patients often look to digital tools freely available on the internet to address their unmet health and information needs. This study identified several ways by which both for-profit and not-for-profit platforms generated income. These income-generating processes can have important implications for the health of patients and incur costs to the health care system, which will be discussed in detail later.

### Advertising

Besides health information, patient platforms can act as a channel to deliver advertisements directly to patients. Advertisements were justified by platforms as necessary for the provision of digital tools to patients without charging up-front fees. Although platforms might claim that users have the choice to ignore advertisements, the labeling of advertisements does not necessarily negate the risks of increased costs. In previous studies, advertisements sent directly to patients, referred to as *direct-to-consumer advertising*, have been associated with increased health care costs because pharmaceutical companies tend to promote more expensive drug therapies (eg, new drugs, new formulations of existing drugs, or novel devices) [20,21]. This may also lead to an increased number of doctor visits or diagnostic testing. For instance, Layton et al [22] illustrated how increased television exposure to testosterone advertisements was associated with the ordering of more testosterone tests and prescribing of more testosterone without testing. Advertising through patient platforms poses unique challenges for many countries that are different from other, more traditional, media outlets. Advertisements posted on the internet from countries with less stringent advertising policies, such as the United States and New Zealand, can reach patients in countries where direct-to-consumer advertising is prohibited [23]. This study adds to the body of literature calling for changes to how advertising and marketing of drugs are regulated globally, as digital tools freely available on the internet enable new channels for advertisements to reach patients across geographic boundaries [24,25].

### Sponsored Tools

Various funding arrangements, such as educational grants or sponsorship, have been described as a beneficial way for patients to access tools without paying up-front fees. Whether these tools may be beneficial to patients depends on a number of factors, including the quality of information delivered. Future research is needed to better understand the role that commercial influences might play in the accuracy of the health information provided, which can add to the body of literature on the quality of consumer health information on the internet [26,27]. This study illustrates the possibility of posting editorial content on patient platforms written to support platform sponsors. Moreover, acknowledging corporate sponsorships could encourage individuals to act more favorably toward sponsors in future purchases of products or services. For instance, Jong et al [28] studied the impact of a national campaign to raise awareness of onychomycosis (toenail fungal infection) in Denmark. Despite the availability of various antifungal treatment options, the campaign was followed by an increase in prescriptions for the drug sold by the campaign sponsor only but not for other antifungal drugs for onychomycosis.

In addition, this study identified tools offered by not-for-profit platforms in partnership with sponsors or partners, which may be motivated to raise awareness for rare diseases or specific types of cancers. Although some patients could potentially benefit from earlier detection or treatment of a rare disease or cancer, the full impact of disease awareness tools needs further studies. For example, Mailankody and Prasad [29] raised concerns over a disease awareness campaign for a rare disease (polycythemia vera) that was sponsored by a pharmaceutical company that sells a drug to treat the rare condition. In their view, increased awareness of the rare condition and the drug therapy to treat it could increase the number of patients diagnosed with the condition. However, the diagnostic criteria for the rare disease have not yet been clearly defined. Thus, the authors expressed concerns over the potential misuse of the drug beyond the indications studied and approved by the FDA. They argued that such efforts could drive “wasteful diagnostic testing, overdiagnosis and inappropriate therapy” [29].

As with disease awareness, we identified diagnostic or self-assessment tools provided directly to patients, including tools sponsored by pharmaceutical companies that sell drugs for treating the conditions. Although some patients could potentially benefit from earlier diagnosis, the impact of patients seeking drug therapies based on self-assessments is not known and could potentially cause harm [30-32]. For example, people can ask their physicians to prescribe drug therapies for conditions determined by self-assessments, attempt to obtain these medications directly from internet-based drug distributors (operating from global jurisdictions) [33,34], or order diagnostic tests online [35]. Therefore, this study highlights the need for more research to gain a better understanding of the benefits and risks and patient behaviors associated with the use of sponsored tools, including self-ordering of diagnostic tests and/or drug therapies.

### New Ways to Reach Patients

This study identified how marketing services offered by patient platforms could potentially influence the way patients manage their health issues, including the choice of drug therapies or diagnostic tests or enrollment in clinical trials. For instance, coupons offered to help users save money can potentially lead to changes in drug therapies. Mintzes et al [36] raised concerns over pharmaceutical marketing efforts to encourage patients to shift drug use to marketed drug therapies, which may be more expensive. We also found that some platforms connect patients directly with pharmaceutical companies for clinical trials. However, the impact of such direct recruitment is largely unknown, as it deviates from the traditional process where patients consult with their health care provider for optimal therapy. Although some patients could potentially benefit from receiving information from site sponsors (such as pharmaceutical companies), this study highlights the need for more research to better understand the impact of processes that might eliminate traditional intermediaries, such as health care providers who help patients interpret technical information and make informed decisions.

### Sharing User Information

This study found numerous examples where user information can be shared with sponsors, which operate for-profit platforms. In one case, the platform was transparent in warning users that their information could be sold to companies (eg, insurance companies) and the associated potential risks. Other platforms were not as transparent. As digital tools, including patient platforms, apps on mobile devices, and wearables, play an increasingly important role in defining what health data are being collected, accessed, and subsequently used for improving health outcomes, there is a critical need for more research and governance in this evolving area.

### Conclusions and Policy Implications

In summary, this study revealed many mechanisms by which patient platforms generate income to support their operations, gain profit, or both. Although these income-generating processes can occur on social media in general, patient platforms are health-specific. Thus, they can have health implications on patients and financial impacts on patients and the health care system. Although digital tools may be mutually beneficial to patients, platform owners or operators, sponsors, and partners (eg, enrollment in clinical trials or services that would not otherwise be available to patients), in other cases, they may have negative consequences and potentially harm patients. Given that direct access to platform tools may serve as a way for many patients to gain control over their health issues, there is a need to improve how patients are informed about the risks and benefits of using digital tools freely available on the internet.

Besides transparency in disclosing income-generating processes, there is a broader question as to whether all patients have the capacity to understand the information provided. For instance, it has been reported that patients often lack *the capacity to obtain, understand, and act upon health information and services to make appropriate health decisions on their own* [37] and that improving health literacy is associated with improving patient engagement [38]. Thus, broader system changes may be needed to increase health literacy at the population level [39,40]. Nevertheless, to ensure that patients can more thoroughly understand and make informed choices about the information and behaviors that these health-specific websites are marketing to them, there is a need for health authorities to expand their role to the evolving digital health landscape [41]. As new digital technologies change how we access and use health data, this study evokes the policy issue of whether and how authorities should protect users from processes that may not be in their best interests.

### Limitations and Future Research

Although the collection of publicly available data from patient platforms provided a rich source of information about what platforms were doing, there are some limitations to this study and opportunities for future research. First, we analyzed platforms only but not the behavior of platform users. Future research is needed to explore how people actually use different types of digital tools and their impact on health utilization and expenditures. Second, although the platform selection process was designed to capture the variability of platforms across several attributes, our findings are not representative of all patient platforms, particularly because we only explored English websites (in the United States and Canada). Future studies can explore platforms in other languages or target other patient-important outcomes or dimensions, for example, different cultural values or norms, conditions with high cost, or lack of effective treatment options, which might offer other opportunities for income-generating processes. Third, our findings reflect processes occurring on the selected platforms during the data collection period only (September 2013 to August 2014). We acknowledge that the digital world has evolved since the time of our data collection and analysis. Further, we notice that many general social media platforms have updated their terms of use and privacy policy in light of recent events such as the Facebook or Cambridge Analytica scandal [42]. Similar changes may have occurred in patient platforms’ policies. Nevertheless, this study captures a snapshot of the platforms at the time of data collection, which offers a useful way to analyze what can happen. Our findings highlight critical policy implications for patients, providers, and policy makers that continue to be important in our digital health world. Finally, we did not assess the quality of information provided by the digital tools, which could affect whether their business models might offer a win-win situation. Given the current infodemia (or epidemiology of misinformation), where *accurate and timely knowledge translation* can be distorted by various factors such as political and commercial influences [43], and that *fake news* may be distributed or even generated (eg, using artificial intelligence) [42], future research to assess the quality of information on patient platforms will be beneficial. Related to this point, we described our analysis that focused on the written content on business models. Future research can expand our understanding of both the income-generating processes and transparency of disclaimers by exploring the visual presentation of content and the prominence of the placement of the messages on the site. Nevertheless, our findings point to important policy implications in the growing area of online resources provided to patients directly, particularly because platforms are not currently governed to ensure the sharing of accurate information or information that does not do harm.

## Abbreviations

MS: multiple sclerosis
FDA: Food and Drug Administration
